# Evaluation of viral co-infections among patients with community-associated *Clostridioides difficile* infection

**DOI:** 10.1371/journal.pone.0240549

**Published:** 2020-10-19

**Authors:** Lauren Korhonen, Jessica Cohen, Nicole Gregoricus, Monica M. Farley, Rebecca Perlmutter, Stacy M. Holzbauer, Ghinwa Dumyati, Zintars Beldavs, Ashley Paulick, Jan Vinjé, Brandi M. Limbago, Fernanda C. Lessa, Alice Y. Guh

**Affiliations:** 1 Division of Healthcare Quality Promotion, Centers for Disease Control and Prevention, Atlanta, GA, United States of America; 2 Atlanta Research and Education Foundation, Atlanta, Georgia, United States of America; 3 Division of Viral Diseases, Centers for Disease Control and Prevention, Atlanta, Georgia, United States of America; 4 Emory University School of Medicine, Atlanta, Georgia, United States of America; 5 Veterans Affairs Medical Center, Atlanta, Georgia, United States of America; 6 Maryland Department of Health, Baltimore, Maryland, United States of America; 7 Minnesota Department of Health, St Paul, Minnesota, United States of America; 8 Career Epidemiology Field Officer Program, Centers for Disease Control and Prevention, Atlanta, Georgia, United States of America; 9 New York Emerging Infections Program and University of Rochester Medical Center, Rochester, New York, United States of America; 10 Oregon Health Authority, Portland, Oregon, United States of America; University of Minnesota Twin Cities, UNITED STATES

## Abstract

We assessed viral co-infections in 155 patients with community-associated *Clostridioides difficile* infection in five U.S. sites during December 2012–February 2013. Eighteen patients (12%) tested positive for norovirus (n = 10), adenovirus (n = 4), rotavirus (n = 3), or sapovirus (n = 1). Co-infected patients were more likely than non-co-infected patients to have nausea or vomiting (56% vs 31%; p = 0.04), suggesting that viral co-pathogens contributed to symptoms in some patients. There were no significant differences in prior healthcare or medication exposures or in CDI complications.

## Introduction

*Clostridioides difficile*, a bacterial gastrointestinal pathogen, is the most common cause of antibiotic-associated diarrhea. Although primarily a healthcare-associated infection, *C*. *difficile* infection (CDI) has been increasingly reported among persons in the community without traditional CDI risk factors. Studies have shown that >35% of patients with community-associated (CA) CDI did not report any recent antibiotic use [1, 2], and >50% of these patients reported nausea or vomiting [2, 3], neither of which is traditionally associated with CDI, raising concerns that some symptoms among patients with CA-CDI might be caused by other pathogens. Previous studies have indicated that viral gastrointestinal infections might be common in children co-infected with CDI [4, 5]. *C*. *difficile* has also been detected among adults during viral gastrointestinal outbreaks in healthcare settings [6, 7]. Although the availability of molecular multiplex panels has led to increased reporting of co-infections among patients with CDI, most published data have not differentiated between community- and healthcare-associated disease [5, 8–11]. In addition, limited data exist regarding the clinical manifestations and disease severity of co-infected compared to non-co-infected CDI patients. The few studies that have compared these two groups of patients have been single-center studies [4, 8]. Therefore, we conducted a multisite analysis to assess the frequency of co-infection with selected viral gastrointestinal pathogens that are commonly associated with nausea and vomiting among patients with known CA-CDI. We also compared the clinical characteristics between co-infected and non-co-infected CA-CDI patients.

## Materials and methods

The Centers for Disease Control and Prevention’s (CDC) Emerging Infections Program (EIP) conducts population- and laboratory-based CDI surveillance [12]. Five of the EIP sites participated in this project (GA, MD, MN, NY, and OR). The Health Commissioner of the MN Department of Health had made CDI reportable within the surveillance catchment area, which meant that CDI surveillance was deemed a public health activity and did not require review by the MN Institutional Review Board (IRB). The IRBs of the other participating EIP sites (Emory University, Atlanta Veteran Affairs, Georgia Department of Public Health, Maryland Department of Health, New York State Department of Health, Rochester General Health System, and Multnomah County Health Department) and the CDC Human Research and Protection Office had reviewed the surveillance protocol and either deemed it exempt from IRB review or non-research or provided IRB approval with a waiver of informed consent. The EIP investigators had access to personally identifiable information in patient records (M.M.F., R.P., S.M.H., G.D., and Z.B.). Patient names and addresses were removed before any data were shared with CDC investigators.

CDI cases were identified through laboratory reporting during December 2012–February 2013 in select counties in 5 U.S. states (Georgia, Maryland, Minnesota, New York, and Oregon). An incident CA-CDI case was defined as a *C*. *difficile*-positive stool test (toxin or molecular assay) collected as an outpatient or within 3 days of hospitalization from a catchment-area resident aged >2 years with no positive test in the prior 8 weeks and no documented overnight stay in a healthcare facility in the prior 12 weeks. Cases were excluded from this analysis if there was no documentation of diarrhea or if stool specimens were not available for testing.

EIP staff performed medical-record abstraction for demographic data, comorbidities, relevant risk factors based on prior epidemiological studies, and clinical characteristics. The occurrence of death following CDI diagnosis was obtained from the state death registries. The Chi-square test or Fisher exact test (where applicable) was used to compare co-infected and non-co-infected cases.

Stool specimens from cases meeting inclusion criteria were cultured for *C*. *difficile* in 2012–2013 by either the Minnesota Department of Health Public Health Laboratory or CDC. Recovered *C*. *difficile* isolates underwent strain typing at CDC using capillary-based PCR-ribotyping; results were analyzed against a library of standard profiles using BioNumerics software (Applied Maths, Austin, TX). All stool specimens were tested at CDC for norovirus, rotavirus, sapovirus, astrovirus and adenovirus by real-time (RT)-PCR.

## Results

Of 528 CA-CDI cases identified, 155 had documented diarrhea and stool available for testing. Among the 155 cases tested, 18 (12%) were co-infected with norovirus (n = 10), adenovirus (n = 4), rotavirus (n = 3), or sapovirus (n = 1); astrovirus was not detected in any of the stool specimens. No co-infected cases had more than one viral co-infection. A similar proportion of co-infected and non-co-infected cases were positive for *C*. *difficile* by toxin enzyme immunoassay (EIA) (39% vs 34%; p = 0.70) (Table 1).

**Table 1:** Comparison of demographics, prior healthcare and medication exposures, and clinical characteristics between co-infected and non-co-infected community-associated *Clostridioides difficile* infection cases.

Characteristics	Co-infected cases (N = 18) No. (%)	Non-co-infected cases (N = 137) No. (%)	*P*-value
Age group			0.81
2–17 years	1 (6)	8 (6)	
18–44 years	5 (28)	33 (24)	
45–64 years	5 (28)	54 (39)	
≥65 years	7 (39)	42 (31)	
Male sex	11 (61)	50 (37)	0.04
White race	12 (67)	83 (61)	0.62
*C*. *difficile* diagnostic assay			
Toxin EIA positive	7 (39)	47 (34)	0.70
Toxin EIA negative but molecular assay positive	6 (33)	33 (24)	0.40
Molecular assay positive (Toxin EIA not performed)^a^	5 (28)	57 (42)	0.26
*C*. *difficile* cultured from stool	10 (56)	124 (91)	<0.0001
Most common ribotypes identified^b^			
027	0/10 (0)	21/124 (17)	0.36
020	3/10 (30)	10/124 (8)	0.06
106	1/10 (10)	10/124 (8)	0.59
078	1/10 (10)	7/124 (6)	0.47
002	0/10 (0)	7/124 (6)	1.00
Nausea or vomiting	10 (56)	43 (31)	0.04
Charlson comorbidity index ≥1	11 (61)	62 (45)	0.21
Prior outpatient healthcare exposures^c^			
Any outpatient exposure^d^	3 (17)	41 (30)	0.28
Dialysis	0 (0)	2 (1)	1.00
Surgical procedure	0 (0)	6 (4)	1.00
Emergency department visit	3 (17)	32 (23)	0.77
Observation unit stay	0 (0)	3 (2)	1.00
Prior medication exposures^c^			
Any antibiotics	9 (50)	79 (58)	0.54
Cephalosporins	3 (17)	17 (12)	0.71
Fluoroquinolones	0 (0)	15 (11)	0.22
Proton pump inhibitors	9 (50)	39 (28)	0.06
Immunosuppressants	4 (22)	26 (19)	0.75
CDI complications^e^	0 (0)	8 (6)	0.60
Died within 30 days of CDI diagnosis	0 (0)	2 (1)	1.00

Abbreviations: EIA, enzyme immunoassay; CDI, *Clostridioides difficile* infection

^a^Toxin EIA results were not available because these laboratories only utilized a molecular assay for *C*. *difficile* testing.

^b^The top 5 most common ribotypes identified from the total sample of *C*. *difficile* isolates were included in the table. Among the 10 co-infected cases with available *C*. *difficile* isolates for strain typing, 8 different ribotypes were identified; among 124 non-co-infected cases with available *C*. *difficile* isolates, 44 different riboypes were identified.

^c^Exposure period was during the 12 weeks preceding the date of *C*. *difficile*-positive stool collection.

^d^Outpatient exposures only include dialysis, surgical procedure, emergency department visit, and observation unit stay.

^e^CDI complication was defined as having ileus, toxic megacolon, or colectomy.

Co-infected cases were more likely than non-co-infected cases to have nausea or vomiting within one day before or after stool collection (56% vs 31%; p = 0.04) and to be male (61% vs 37%; p = 0.04) (Table 1). Among the 10 co-infected cases with nausea or vomiting, six were tested by toxin EIA for *C*. *difficile*, of whom one was toxin-positive by EIA and the remaining five were positive only by a molecular assay; four were only tested by molecular assay. There were no statistically significant differences in the age distribution and the frequency of prior outpatient healthcare and antibiotic exposures, clinical complications (defined as ileus, toxic megacolon, or colectomy), and crude 30-day mortality between co-infected and non-co-infected cases.

*C*. *difficile* was cultured from the stool specimens of 124 (91%) non-co-infected cases compared to 10 (56%) co-infected cases (p<0.0001) (Table 1). *C*. *difficile* ribotype 027 was only detected among non-co-infected cases (17% vs 0%; p = 0.36).

## Discussion

We found the prevalence of co-infection with viral gastrointestinal pathogens among this sample of adult and pediatric patients with CA-CDI was 12%, with norovirus being the most commonly detected co-pathogen. Another U.S. CDI study of viral co-infections conducted within a year before our study and only among pediatric patients reported a higher prevalence of 24%; similar to our study, norovirus was also the most common virus detected [4].

Other U.S. CDI studies that assessed for any type of gastrointestinal co-pathogen included both community- and healthcare-associated cases and reported co-infection rates ranging widely from 16% to 71% [5, 8–11]. These studies used either a multiplex molecular panel or standard laboratory methods to detect a broader array of gastrointestinal pathogens, including bacteria and parasites. Despite testing for more pathogens than we did, norovirus was still the most common co-pathogen identified in at least three of the studies [5, 9, 10]. However, one of these studies had a small sample of only seven CDI cases [10], limiting its generalizability, and three other studies included children <2 years of age [5, 9, 11], who can have high prevalence of *C*. *difficile* colonization [13]. In contrast, our analysis used data from 155 patients over 2 years of age across 5 geographically-diverse sites.

In other countries, the prevalence of co-infection with a gastrointestinal pathogen among patients with CDI have varied from 34% to 67% [14–16]. In a multicenter study on community-acquired gastroenteritis conducted in 10 European countries, the proportion of CDI patients with co-infection was highest in those <5 years of age [14]. Interestingly, in some countries, bacterial co-pathogens were frequently detected, comprising at least half or more of the co-infections among CDI patients [14, 15].

We found that co-infected cases in our sample of patients were more likely to have nausea or vomiting than non-co-infected cases, though neither of these symptoms are traditionally associated with CDI. In one pediatric CDI study, children with viral co-infections had higher burden of *C*. *difficile* compared to non-co-infected children, but the two groups were clinically indistinguishable [4]. It is possible that some of our CA-CDI cases that tested positive for a viral pathogen were truly co-infected and the viral pathogen caused the nausea or vomiting while the *C*. *difficile* caused the diarrhea. Another study that assessed for additional gastrointestinal symptoms, including abdominal pain and gas, found that patients co-infected with norovirus and CDI had increased severity of gastrointestinal disease symptomatology [17]. Notably, we did not find any differences in the frequency of CDI complications and clinical outcomes between co-infected and non-co-infected cases, consistent with previous reports [4, 8].

Alternatively, some of the co-infected cases in our sample could have been colonized with *C*. *difficile* and infected with only the viral pathogen, since co-infected cases had a lower *C*. *difficile* recovery rate from culture, and most of the co-infected cases with nausea or vomiting that were tested for *C*. *difficile* using toxin EIA were negative by this test; both a low *C*. *difficile* recovery rate and negative toxin EIA results are more common in patients with colonization than those with active infection. Interestingly, however, nausea or vomiting was present in one-third of non-co-infected patients, suggesting these symptoms might also be common in CA-CDI or that they might be caused by some other unidentified gastrointestinal pathogen.

Although we only tested for viral co-pathogens, we reviewed medical records for other bacterial enteric pathogens detected at the time of the CDI diagnosis. Of 116 cases with test results available, only one was positive for *Campylobacter*. Our evaluation was performed on specimens collected during 2012–2013, and it is possible the prevalence of viral co-infections among patients with CA-CDI could have changed since then. Documentation in medical records could have been incomplete, limiting our ability to assess relevant risk factors and additional clinical characteristics. Our analysis was based on a convenience sample of cases, which may not be representative of all CA-CDI patients.

As the use of multiplex molecular panels increases, a greater frequency of co-pathogens might be identified among patients with CDI. A better understanding of the clinical significance of such findings is needed to guide patient management and infection prevention.
